# One-Step Solution Deposition of Antimony Selenoiodide Films via Precursor Engineering for Lead-Free Solar Cell Applications

**DOI:** 10.3390/nano11123206

**Published:** 2021-11-26

**Authors:** Yong Chan Choi, Kang-Won Jung

**Affiliations:** Division of Energy Technology, Daegu Gyeongbuk Institute of Science & Technology (DGIST), Daegu 42988, Korea; kw.jung@dgist.ac.kr

**Keywords:** antimony selenoiodide, SbSeI, solution process, solar cells, one-step method

## Abstract

Ternary chalcohalides are promising lead-free photovoltaic materials with excellent optoelectronic properties. We propose a simple one-step solution-phase precursor-engineering method for antimony selenoiodide (SbSeI) film fabrication. SbSeI films were fabricated by spin-coating the precursor solution, and heating. Various precursor solutions were synthesized by adjusting the molar ratio of two solutions based on SbCl_3_-selenourea and SbI_3_. The results suggest that both the molar ratio and the heating temperature play key roles in film phase and morphology. Nanostructured SbSeI films with a high crystallinity were obtained at a molar ratio of 1:1.5 and a temperature of 150 °C. The proposed method could be also used to fabricate (Bi,Sb)SeI.

## 1. Introduction

Ternary chalcohalides of antimony and bismuth (MChX, where M = Sb, Bi; Ch = S, Se; X = I, Br, Cl) have recently emerged as potential candidates for lead-free solar cell applications because of their promising optoelectronic properties, high stability, low toxicity, and earth-abundant constituents [1,2,3,4]. Solar cells based on these materials are expected to exhibit high device performance because of the ns^2^ electronic configuration of Sb^3+^/Bi^3+^, such as Pb^2+^ in Pb perovskites, which enables defect-tolerant features. These features make them attractive alternatives to Pb perovskites, which are being widely studied in terms of their use in next-generation solar cells. However, the highest device efficiency of solar cells using ternary chalcohalides that has been reported so far is less than 5% [5], which is far below that of Pb perovskites solar cells (>25%) [6,7], and little work has been conducted to date on improving their efficiency. Further work is therefore required to improve device performance. However, fabrication methods for both obtaining and controlling the properties of high-purity chalcohalides suitable for solar cell applications are lacking.

Sb/Bi chalcohalides have been fabricated for solar cell applications using various methods [2]. Among these, the two-step solution-phase method has been demonstrated to be effective in the fabrication of various materials [5,8,9,10,11,12]. In this approach, chalcohalides are obtained via the conversion of chalcogenides formed in the first step of the two-step method, and so the compositions of the final products can be controlled depending on the chalcogenide and halide species used in the first and second steps, respectively. To date, various materials, such as SbSI [8,10], (Sb,Bi)SI [9], BiSI [11], SbSeI [5], and Sb(S,Se)I [12], have been fabricated using this method. In addition, an efficiency of ~4.1% was obtained using this method from solar cells based on antimony selenoiodide SbSeI [5]. Despite remarkable progress, however, this method has limitations when it comes to obtaining a pure-phase film. To form a pure phase, all chalcogenides formed in the first step must react with halides during the second step. However, chalcogenides often cannot react with halides because of their undesirable morphology, leaving a portion of the chalcogenides unconverted in the final product. For example, we have previously found that the intertwined Bi_2_S_3_ morphology formed in the first step prevents the BiI_3_ solution from reaching a deeper region near the bottom, leaving unreacted nanostructures [11]. This problem may be addressed by applying a one-step method based on the precursor solution. To this end, the precursor solution must be designed to form the desired single phase. In order the achieve this, first we selected the chalcohalide SbSeI, and then we began to prepare a precursor solution for it.

Due to the fact that SbSeI belongs to the SbSI family [13], SbSeI formation may be expressed by the following chemical reaction, similar to the case of SbSI [8,10,12,13]: Sb_2_Se_3_ + SbI_3_ → 3SbSeI. In addition, according to the Sb-Se-I phase diagram [14,15], the SbSeI phase is formed through a competing process of two phases, Sb_2_Se_3_ and SbI_3_, under controlled molar ratios and temperature conditions. These results suggest that the control of Sb_2_Se_3_ and SbI_3_ is a key factor in the development of a method for SbSeI fabrication. As a first step, we explored different solutions that could produce Sb_2_Se_3_ and SbI_3_. As a result, we found that two solutions based on SbCl_3_-SeU (Sol A, where SeU is selenourea) and SbI_3_ (Sol B) may be used to form Sb_2_Se_3_ and SbI_3_ phases, respectively, at a low temperature of 150 °C (as shown in Figure S1 of the Supplementary Material). Based on these findings, we developed a solution-processing method for the fabrication of SbSeI thin films. Specifically, we designed a precursor solution that may be used to produce a pure-phase film in a single step by mixing the two solutions. 

In this work, we report a facile one-step solution-processing method based on precursor engineering using Sol A and Sol B solutions. The precursor solutions were synthesized by mixing Sol A and Sol B at different molar ratios. This controlled molar ratio allowed for the manipulation of Sb_2_Se_3_ and SbSeI phases, leading to the formation of a pure SbSeI film under specific conditions. Moreover, the pure phase was obtained at a low temperature of 150 °C. We also applied this approach to the fabrication of other selenoiodides, namely (Bi,Sb)SeI, to prove the versatility of the proposed method in terms of the preparation of various chalcoiodides for solar cell applications.

## 2. Materials and Methods

### 2.1. Chemical and Materials

Antimony (III) chloride (SbCl_3_, 99+ %), antimony (III) iodide (SbI_3_, 99.999%), SeU (NH_2_CSeNH_2_, 99.97%), *N*-methyl-2-pyrrolidinone (NMP, C_5_H_9_NO, anhydrous, 99.5%), and *N*,*N*-dimethylformamide (DMF, HCON(CH_3_)_2_, anhydrous, 99.8%) were purchased from Alfa Aesar (Seoul, Korea). Cadmium sulfate hydrate (CdSO_4_·8/3H_2_O, ≥99.0%), thiourea (TU, NH_2_CSNH_2_, ≥99.0%), and bismuth (III) iodide (BiI_3_, 99%) were purchased from Sigma-Aldrich (Seoul, Korea). Ammonium hydroxide solution (NH_4_OH, 28% NH_3_ in H_2_O) was purchased from Junsei (Tokyo, Japan). All chemicals were used as received without further purification. FTO glass with a sheet resistance of 15 Ω sq^−1^ was purchased from Pilkington (AMG, Yongin-si, Korea). 

### 2.2. Preparation of the CdS/FTO Substrate

A 50 nm-thick CdS layer was deposited on the FTO glass using a chemical bath deposition method. CdS deposition was performed according to a previously reported procedure [16], in which FTO glass was dipped in an aqueous solution containing CdSO_4_·8/3H_2_O, NH_4_OH, and TU. During immersion, the temperature and pH of the solution were maintained at 65 °C and 11–11.5, respectively. After being dipped for 12 min 30 s, the glass was removed from the solution and washed with deionized water several times, before being dried. The sample was then immediately transferred into an N_2_-filled glove box with a moisture control system, in which the H_2_O level was maintained below 1 ppm, to anneal it in an inert gas. Finally, the CdS/FTO substrate was obtained after heating at 400 °C for 1 h in the glove box. 

### 2.3. Synthesis of Precursor Solution and Deposition of SbSeI Thin Films

**Figure 1:**
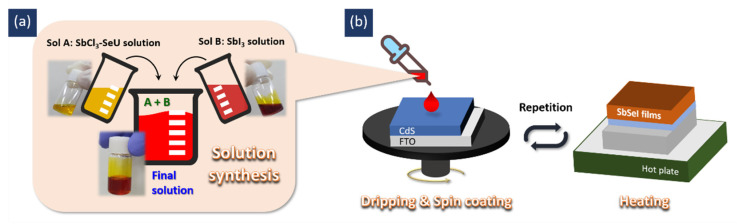
Schematic illustrations of (**a**) the synthesis of the one-step solution, and (**b**) the deposition process for SbSeI thin films.

The precursor solution was synthesized by mixing two stock solutions, Sol A and Sol B, as shown in Figure 1a. In order to synthesize Sol A, 0.5 mmol SbCl_3_ and 1.25 mmol SeU were dissolved in 1 mL of DMF. Sol B was prepared by dissolving 0.5 mmol SbI_3_ in 1 mL of NMP. After stirring the two solutions for 1 h, they were mixed at different molar ratios and stirred for a further 1 h to prepare the precursor solution. After this step, 180 μL of the final solution was spin-coated at 5000 rpm on the pre-cleaned CdS/FTO substrate, followed by heating at 150 °C for 5 min (Figure 1b). This process was repeated five times, and all procedures were performed in a glove box. During solution synthesis, the CdS/FTO substrate was cleaned by UV/O_3_ treatment for 20 min outside the glove box, before being immediately returned to the glove box prior to spin-coating.

### 2.4. Characterization

Optical absorption was measured using a UV-VIS absorption spectrophotometer (Shimadzu UV-2600) in the wavelength range of 400–1200 nm. A sample crystal structure was measured using an X-ray diffractometer (Malvern Panalytical Empyrean, Malvern, UK) in the θ/2θ scan mode. The phase quantification was performed by the Rietveld method using X’Pert HighScore Plus (version 3.0.0) software. A field emission scanning electron microscope (Hitachi S-4800, Tokyo, Japan) was used to investigate sample morphology. Electronic structure was investigated by ultraviolet photoelectron spectroscopy (UPS) using an X-ray photoelectron spectrometer (Thermo Scientific ESCALAB 250Xi, Lexington, MA, USA). 

## 3. Results and Discussion

**Figure 2:**
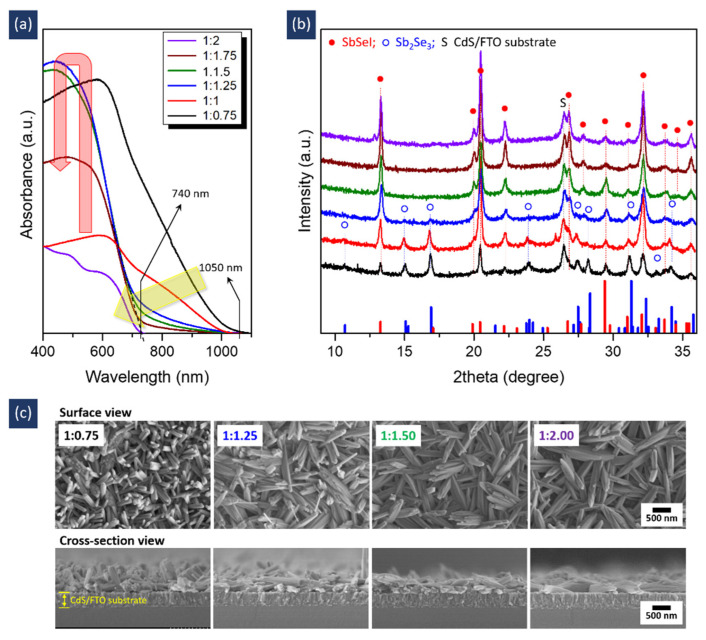
(**a**) Absorption spectra, (**b**) XRD patterns, and (**c**) FESEM images of samples prepared using precursor solutions with different Sol A:Sol B molar ratios. The peak positions of Sb_2_Se_3_ and SbSeI are shown in (**b**), based on the reference data of Sb_2_Se_3_ (ICDD # 98-065-1518, blue column) and SbSeI (ICDD # 98-003-1292, red column).

The precursor solution was synthesized by mixing Sol A and Sol B, and so it was expected that the molar ratio of Sol A and Sol B would affect film formation. To verify this hypothesis, we investigated the absorption properties, crystalline structures, and morphologies of films fabricated using precursor solutions with different Sol A:Sol B molar ratios. At a ratio of 1:0.75 (denoted as a black line in Figure 2a), an absorption edge of ~1050 nm, which is consistent with the value of Sb_2_Se_3_ [17,18], was observed. The sample also exhibited a dominant Sb_2_Se_3_ phase (ICDD # 98-065-1518), as shown in the X-ray diffraction (XRD) pattern (Figure 2b, Tables S1 and S2). Concerning the morphology shown in the field emission scanning electron microscopy (FESEM) image of Figure 2c, nanorods with a diameter of ~50 nm were found to grow randomly on the substrate. These results indicate that Sb_2_Se_3_ nanorods were mainly formed under these conditions. As the SbI_3_ content increased to 1:1.5, the absorption edge shifted toward a wavelength of 740 nm (bandgap *E*_G_ of 1.68 eV), corresponding to the value for SbSeI [5], as indicated by the yellow arrow in Figure 2a. The absorption intensity in the short-wavelength region below 740 nm also gradually increased (denoted by a red arrow). In addition, at a molar ratio of 1:1.5, the SbSeI phase (ICDD # 98-003-1292) became dominant, whereas the Sb_2_Se_3_ phase decreased and then disappeared (Figure 2b and Table S1). As the SbI_3_ content increased, the nanorods aggregated to form nanostructures (Figure 2c). Furthermore, the increase in SbI_3_ induced a decrease in absorption intensity, although this did not affect the XRD patterns and morphology. These results imply that a nanostructured SbSeI film with high crystallinity may be fabricated at a specific molar ratio of 1:1.5. 

**Figure 3:**
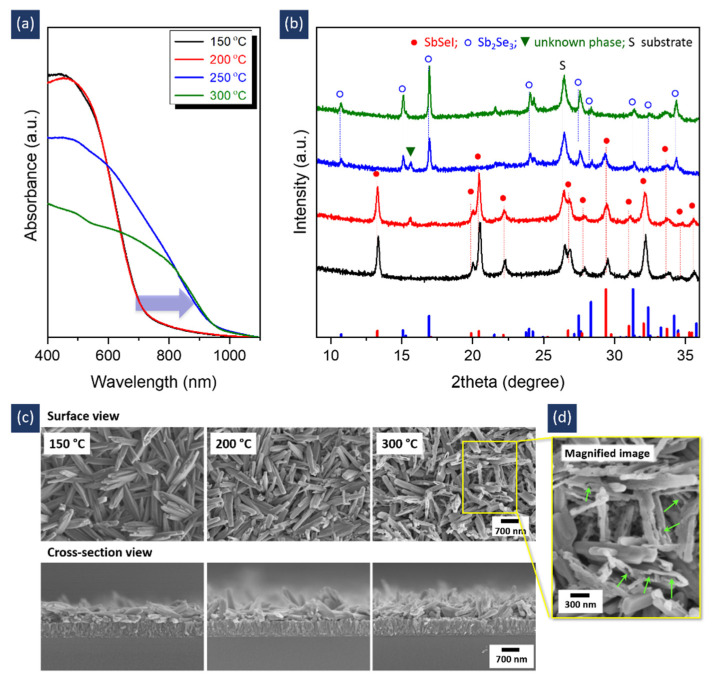
(**a**) Absorption spectra, (**b**) XRD patterns, and (**c**) FESEM images of samples prepared at different annealing temperatures. (**d**) Magnified image, marked by a yellow box in (**c**). All samples were fabricated using the precursor solution with a Sol A:Sol B molar ratio of 1:1.5. In (**b**), two reference patterns of Sb_2_Se_3_ ((ICDD # 98-065-1518) and SbSeI (ICDD # 98-003-1292) are shown as blue columns and red columns, respectively.

In addition to the molar ratio in the precursor solution, we found that annealing temperature played a key role in the formation of pure-phased SbSeI films, as shown in Figure 3. At a temperature of 200 °C, the absorption spectrum was almost equal to that at 150 °C (Figure 3a). However, an unknown peak (the green arrow pointing downwards in Figure 3b) appeared with a decreased SbSeI phase (Figure 3b and Table S3). Further increasing the temperature to 250 °C caused a shift in the absorption edge from 740 nm to 1050 nm (denoted as a blue arrow), revealing a phase change from SbSeI to Sb_2_Se_3_. This change was confirmed by the XRD results (Figure 3b and Table S3), in which the Sb_2_Se_3_ phase predominantly appeared when the temperature was increased to 250 °C. Note that the unknown phase may be considered to be an intermediate Sb-Se-I phase because it is formed in a temperature region where Sb_2_Se_3_ and SbSeI phases can coexist. When the temperature reached 300 °C, only XRD peaks corresponding to the Sb_2_Se_3_ phase were observed. The morphology was very similar to that at 150 °C, as shown in Figure 3c, although several voids were observed in the nanostructures, as indicated by the green arrows in the magnified image (Figure 3d). This similarity in morphology suggests that SbSeI was formed at an early stage during formation at 300 °C. However, because the SbSeI phase is unstable at a higher temperature and is prone to decomposition [14,15], SbI_3_ may evaporate from the initially formed SbSeI as the reaction proceeds, creating voids in the nanostructures. As a result, Sb_2_Se_3_, which is similar in morphology to SbSeI despite containing many pores, was formed. A low temperature of 150 °C was therefore required to obtain pure SbSeI films. This temperature was also confirmed by an investigation of the XRD pattern in the low-temperature region of 120–180 °C (Figure S2 of the Supplementary Material).

**Figure 4:**
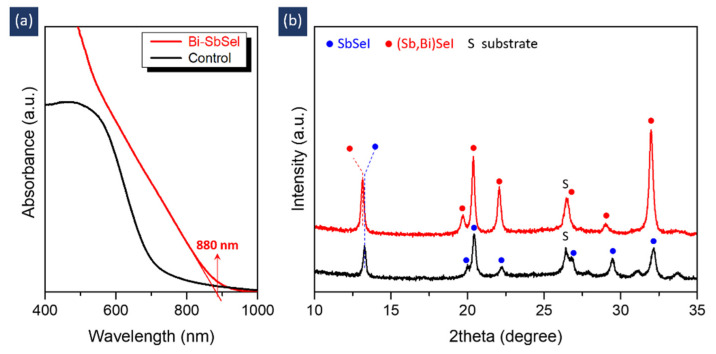
(**a**) Absorption spectra and (**b**) XRD patterns of control and Bi-SbSeI samples.

Given that the phase of the fabricated films was determined by the type of solution used in the precursor solution, it was possible to fabricate various chalcohalides by changing the starting solutions. To verify this possibility, we modified the precursor solution by introducing BiI_3_ instead of SbI_3_ and deposited it on a CdS/FTO substrate following the optimized procedures. For convenience, the sample fabricated using the optimized solution shown in Figure 2 is denoted as ‘control’ in Figure 4. The sample fabricated using the BiI_3_-modified solution is also denoted as ‘Bi-SbSeI’. As shown in Figure 4a, the Bi-SbSeI sample exhibits an absorption edge of ~880 nm, which corresponds to an *E*_G_ of ~1.41 eV. This *E*_G_ value is lower than that of SbSeI (~1.68 eV) but higher than that of BiSeI (~1.32 eV) [19,20]. Figure 4b and Table S4 show that the XRD peaks of Bi-SbSeI are located between the two references for BiSeI (ID: mp-23020, The Materials Project) [21] and SbSeI (ICDD # 98-003-1292). The detected peaks were symmetrical, indicating that a single phase was formed. Based on these results, it can be concluded that a single-phase material composed of (Bi,Sb)SeI was successfully formed by modifying the precursor solution. This suggests that the proposed method can be used to fabricate various chalcohalides, such as SbSI, BiSI, and related alloys.

**Figure 5:**
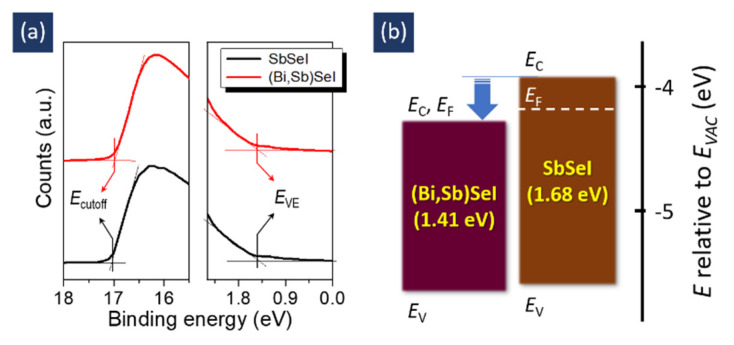
(**a**) UPS spectra of SbSI and (Bi,Sb)SeI, and (**b**) the derived energy level diagram.

We investigated the electronic structures of the samples (shown in Figure 4) further by analyzing their UPS spectra (Figure 5a). We obtained two values from the spectra, one for the cut-off energy, *E*_cutoff_, and another for the valence band edge energy, *E*_VE_. From the two equations *E*_V_ = *E*_F_ + *E*_VE_ and *E*_V_ = hυ − (*E*_cutoff_ − *E*_VE_), we also calculated the following three values: conduction band minimum (*E*_C_), valence band maximum (*E*_V_), and Fermi level energy (*E*_F_) [22]. These values are listed in Table S5 of the Supplementary Material and are shown in Figure 5b. The (Bi,Sb)SeI sample presented a similar *E*_V_ to SbSeI, but a lower *E*_C_ value. This result indicates that the incorporation of Bi into SbSeI induces a downshift in *E*_C_. This result also suggests that electronic structure may be controlled via compositional engineering. The proposed method could therefore be applied to optimizing electronic properties in order to render materials suitable for solar cell applications via compositional engineering. However, the current method is limited when it comes to forming a dense film, as shown in Figure 2c. It is generally accepted that morphology plays a key role in obtaining high efficiency [7]. Typically, dense films with a large grain size contribute significantly to achieving high performance. Thus, it can be deduced that it is very difficult to obtain a high level of efficiency from our films. A preliminary result confirmed that the device exhibited a very poor efficiency of 0.23% (Table S6 of the Supplementary Materials). Therefore, we are currently adapting the proposed method in order to both improve and optimize the morphology of SbSeI films. The morphology may be improved by various approaches, such as annealing optimization, post-treatment, the use of additive effects, and solvent annealing [7].

## 4. Conclusions

A simple one-step solution-processing method for the fabrication of SbSeI films is presented. The pure-phase SbSeI film (*E*_G_ = 1.68 eV) was obtained at a low temperature of 150 °C using a precursor solution with a molar ratio of Sol A:Sol B = 1:1.5. Modifying the precursor solution resulted in the formation of (Bi,Sb)SeI. These results suggest that the proposed method can be readily applied to the fabrication of various chalcohalides, as well as to optimize their electronic structures in order to render them suitable for solar cell applications. However, further improvements are necessary to optimize key factors, such as morphology, crystalline orientation, and defects, so as to make them suitable for high performance Sb/Bi chalcohalide solar cells. 

## Data Availability

All relevant data supporting the findings of this study are available within the article and its Supplementary Material.

## Supplementary Materials

The following are available online at https://www.mdpi.com/article/10.3390/nano11123206/s1: Figure S1: absorption spectra and XRD patterns of samples fabricated with Sol A and sol B; Table S1: Quantitative ratio of Sb_2_Se_3_ and SbSeI phases; Table S2: Lattice spacings for each phase; Table S3: Quantitative ratio of Sb_2_Se_3_, SbSeI, and unknown phases; Figure S2: XRD patterns of samples fabricated at 120–180 °C; Table S4: Comparison of XRD peaks of (Bi,Sb)SeI with references; Table S5: *E*_cutoff_, *E*_VE_, *E*_C_, *E*_V_, and *E*_F_ values of SbSI and (Bi,Sb)SeI samples; Table S6: Device parameters of the SbSeI solar cells.
