# Supplementary material for: One-Step Solution Deposition of Antimony Selenoiodide Films via Precursor Engineering for Lead-Free Solar Cell Applications
Source: Nanomaterials (Basel). 2021 Nov 26;11(12):3206. doi: 10.3390/nano11123206 (PMC8703622; doi:10.3390/nano11123206)
Supplement: Supplementary file 1 [file nanomaterials-11-03206-s001.zip › nanomaterials-1462528-supplementary.pdf]

Supplementary Material

One-Step Solution Deposition of Antimony Selenoiodide Films via Precursor Engineering for Lead-Free Solar Cell Applications

Yong Chan Choi * and Kang-Won Jung

Division of Energy Technology, Daegu Gyeongbuk Institute of Science & Technology (DGIST), Daegu 42988, Korea; kw.jung@dgist.ac.kr

* Correspondence: ycchoi@dgist.ac.kr

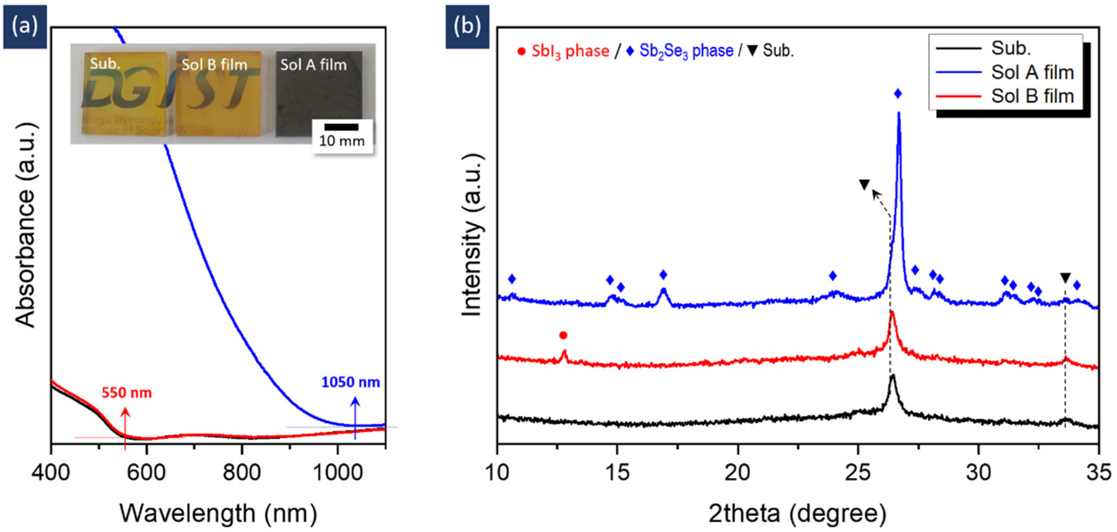

Figure S1. (**a**) Absorption, sample photographs, and (**b**) XRD patterns of samples fabricated using Sol A (blue lines) and Sol B (blue lines). XRD patterns were indexed based on two references for orthorhombic Sb_2Se_3 (ICDD #98-065-1519) and monoclinic SbI_3 (ICDD #98-002-6082). "Sub.," "Sol A film," and "Sol B film" indicate the CdS/FTO substrate, and films fabricated by Sol A and Sol B, respectively. Note that Sol A and Sol B films were annealed at 150 °C in the glove box.

The Sol A film exhibits an absorption edge of ~ 1050 nm (E_G of ~1.18 eV), and its XRD pattern matches that of orthorhombic Sb_2Se_3. The Sol B film exhibits an absorption edge of ~ 550 nm (E_G of ~2.25 eV) and SbI_3 phase. These results indicate that the Sb_2Se_3 and SbI_3 phases were successfully formed using Sol A and Sol B, respectively.

Table S1. Quantitative ratio of Sb_2Se_3 and SbSeI phases, shown in Figure 2b.

Samples	1:0.75	1:1	1:1.25	1:1.5	1:1.75	1:2
Sb_2Se_3 phase	39%	26%	9%	100%	100%	100%
SbSeI phase	61%	74%	91%	0	0	0

Table S2. Lattice spacings for each phase, obtained from the XRD pattern of the 1:1 sample, shown in Figure 2b.

2theta (°)	Phase	Lattice plane	Calculated lattice spacing (Å)
10.72	Sb_2Se_3	(101)	8.24717
13.333	SbSeI	(101)	6.63528
15.035	Sb_2Se_3	(200)	5.88775
16.876	Sb_2Se_3	(201)	5.24947
20.07	SbSeI	(102)	4.42111
20.5133	SbSeI	(200)	4.32613
22.295	SbSeI	(011)	3.98427
24.03	Sb_2Se_3	(103)	3.70014
26.588	SbSeI	(202)	3.34987
27.461	Sb_2Se_3	(302)	3.24538
28.18	Sb_2Se_3	(211)	3.16407
29.549	SbSeI	(112)	3.02062
31.183	Sb_2Se_3	(212)	2.86596
32.185	SbSeI	(301)	2.77897
34.080	Sb_2Se_3	(402)	2.62867

Table S3. Quantitative ratio of Sb_2Se_3, SbSeI, and unknown phases, shown in Figure 3b.

Samples	150 °C	200 °C	250 °C	300 °C
Sb_2Se_3 phase	100%	0	92%	0
SbSeI phase	0	93%	0	0
Unknown phase	0	7%	8%	100%

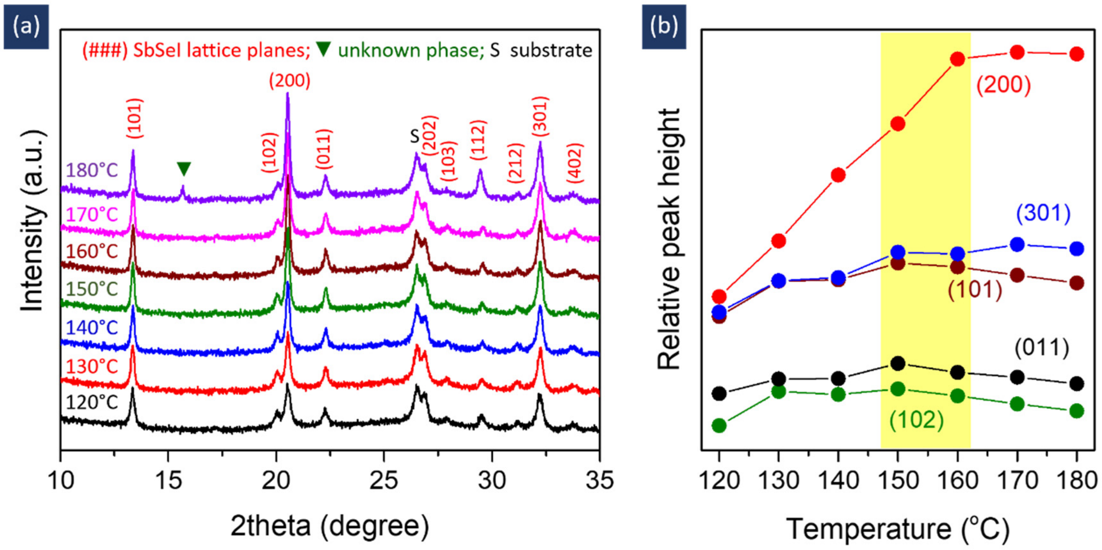

Figure S2. (**a**) XRD patterns of samples fabricated at 120–180 °C and (**b**) corresponding peak height of typical five SbSeI lattice planes. As the temperature increased to 150 °C, all XRD peaks gradually increased, as shown in Figure S2b. However, further increasing the temperature led to a decrease in the peak height. Whereas, the peak height of (200) plane increased until 160 °C and then saturated. In addition, the unknown phase, related to intermediate Sb-Se-I phase, was observed at a high temperature of 180 °C. These results indicate that 150 and 160 °C, highlighted by a yellow box in (b), are proper to form pure SbSeI film.

Table S4. Comparison of five typical XRD peaks of the sample, obtained from the XRD pattern (Figure 4b), with two reference XRD peaks. BiSeI and SbSeI reference data were obtained from ID # mp-23020 (The Materials Project) and ICDD # 98-003-1292, respectively.

Plane	(011) or (101)	(012) or (102)	(020)	(021) or (201)	(120) or (210)
BiSeI reference	12.52	18.58	19.68	21.16	28.73
Sample	13.18	19.71	20.35	22.06	29.05
SbSeI reference	13.27	19.89	20.43	22.17	29.77

Table S5. E_{cutoff}, E_{VE}, E_C, E_V, and E_F values of SbSI and (Bi,Sb)SeI samples.

Samples	E_{cutoff} (eV)	E_{VE} (eV)	E_C (eV)	E_V (eV)	E_F (eV)
SbSeI	17.05	1.42	3.90	5.58	4.17
(Bi,Sb)SeI	16.98	1.42	4.25	5.70	4.25

Table S6. Device parameters of various SbSeI solar cells depending on the film fabrication conditions. For device fabrication, Spiro-OMeTAD and Au (~ 70 nm) were sequentially deposited on the surface of SbSeI films as hole transporting layer and electrode, respectively, according to a previously reported procedure [1]. The results showed that the highest efficiency was obtained from the optimized conditions, i.e. 1:1 molar ratio and 150 °C.

Film fabrication conditions (Molar ratio / annealing temperature)	J_{SC} ($mA \cdot cm^{-2}$)	V_{OC} (mV)	FF (%)	PCE (%)
1:1 / 150 °C	0.15	372.96	42.91	0.02
1:1.25 / 150 °C	0.78	581.03	38.39	0.17
1:1.5 / 150 °C	0.88	633.03	41.71	0.23
1:1.75 / 150 °C	0.68	620.68	40.11	0.17
1:1.5 / 200 °C	0.67	569.25	33.79	0.13
1:1.5 / 250 °C	0.15	356.50	39.32	0.02

J_{SC}: short circuit current density, V_{OC}: open-circuit voltage, FF: fil factor, and PCE: power conversion efficiency.

Reference

[1] Choi, Y.C.; Lee, S.W.; Kim, D.-H., Antisolvent-assisted powder engineering for controlled growth of hybrid $CH_3NH_3PbI_3$ perovskite thin films. *APL Mater*. **2020**, *5*, 026101.
